# Supplementary material for: The intentions of pregnant women to give birth at a health facility and associated factors in the Aleta-Wondo rural District, Ethiopia: A community based cross-sectional study
Source: PLOS Glob Public Health. 2024 May 10;4(5):e0003215. doi: 10.1371/journal.pgph.0003215 (PMC11086849; doi:10.1371/journal.pgph.0003215)
Supplement: Attachment — Submitted filename: Review of PGPH-D-23-02489.docx [file pgph.0003215.s002.docx]

**The intentions of pregnant women to give birth at a health facility and associated factors in the Aleta-Wondo rural District, Ethiopia: A community based cross-sectional study**

Overall comments to the author:

- Significant grammar adjustments necessary for publication, currently tone does match the caliber of journal selected
- Flow seems very “piece-y”
- important to provide additional clarity around methodology and why decisions were made
- need for more specificity and clarity around presented results
- do not present results in the discussion, need to use this section to tie results into context and larger body of existing work
- Need to strengthen conclusion

ABSTRACT

- “out of the respondents…” – repeated 2x
- “having information from health professionals…” – meaning not super clear overall, consider rephrase
- “less than two-thirds of the respondents…” – repetitive, cut or rephrase

INTRODUCTION

- L32/33 – citation needed
- L34 – redundant
- L35 – “…can save nearly three-fourths this death.” Awkward phrasing, needs citation
- L37 – what are childbirth’s “courses”? awkward phrasing and unclear what is meant by this
- L40 – “are influencing” should be changed to “influence” to make the tenses match
- L42 – “despite agreement that…” this is arguably a political stance and is not in fact universal, consider speaking to the context of the study for a claim like this. You have yet to discuss the setting – this should really happen earlier on
- L45/46 – citation needed
- L46/47 – “In fact, need factors can be driven by pregnancy related factors such as perception” this does not make sense
- L47 – don’t use “in fact” twice in a row, rephrase
- L47/48 – “…women’s intentions to involve in utilization of health facility…” awkward grammar
- L51 – “in the world” consider rephrasing to say “worldwide”
- L51 – “highly varying…” consider rephrasing to say “highly variable”
- L52 – “poor” has not yet been defined, would suggest consistent use of WHO/World Bank terminology (e.g. low income, middle income, high income countries)
- L52/53 – suggest combining the first two sentences of this paragraph to be less repetitive and more concise
- L55 – a cause of maternal mortality being “high lifetime risk of maternal mortality” is not a particularly clear concept, consider rephrasing
- L56 – is this in Ethiopia specifically, worldwide or in SSA? Important to specify the context of the information you present as these will not be universal truths
- L56 – “poor health care seeking behavior/pregnant women’s ignorance of receiving skilled care” grammatically is clunky, also reads as quite judgmental and not helpful to the overall argument you’re trying to make, consider a rephrase
- L57 – potentially worth including information about what strategies have been utilized to date to strengthen your discussion
- L58 – “the practice of skilled birth attendance is still in alarming condition..” meaning unclear of this part of the sentence
- L60 – what model are you referencing? This is the first mention of a “model” in the paper, make sure you’re not skipping ahead or using different words to mean the same thing
- L61 – “some people...” too casual a tone for this sentence; overall meaning not clear, consider a rephrase
- L68 – what does this statement mean in context? Expected by whom?
- L71 – why must families consider that “every pregnancy” be at risk of the same complications when we know this isn’t the case? Suggest removing sentence as it does not support your argument
- L75 – delete “so”
- L76 – “yet, far it is not known…” already stated, consider rephrasing for grammar as well
- L78 – suggest providing more information about the exact context and why this was chosen, currently it seems like an afterthought
- Overall commentary: need to make significant grammar adjustments in this section, create more of a flow between ideas ensuring that point a leads to point b and so on, condense information to fit more of a paragraph format rather than one or two sentences at a time

MATERIALS AND METHODS

- L82 – delete “which is”
- L84 – it seems like you’re making a distinction between “female” and “women” which reads a bit odd, suggested rephrase: “…from which approximately 102,054 are female, with 44,039 of reproductive age.”
- L87 – why was it determined that this would be the methodology used? Provide justification in 1-2 sentences
- L87/88 – suggested rephrase “Pregnant women in the Aleta-Wondo rural district of Sidama Regional State were the population under study.”
- L88/89 – suggested rephrase: “Among this population pregnant women with…were included in this study analysis”
- L95 – why was this the assumed p value? I see that you’ve cited a source but not clear why it was chosen, be sure to justify
- L96 – 421 what? You mention pregnant women and you mention kebeles, make sure you’re being very clear
- L97 – important to have defined “kebeles” before this mention
- L97 – how was information about the kebeles collected? Public record, general knowledge, survey, etc – provide clarity
- L127 – suggested rephrase to improve tone “It was then translated back to English by translation specialists to verify the consistency of translation.”
  - Were these individuals part of the research team? Important to appropriately credit sources of support/information
- L129, L131 – not necessary to say “actual data collection,” you can directly say “data collection”
- L131 – did the pre-test verify the efficacy of the tool? Important to speak to the results of the pre-test if you’re going to mention it
- L132/133 – how were these checked?
- L137 – do not capitalize “Socio-demographic and Reproductive”
- 139 – consider rephrase of “…participants’ perceptions of benefits…”
- Overall commentary: need to make significant grammar adjustments in this section, important to provide additional clarity around methodology and why decisions were made

Do we have access to study materials/tools used?

RESULTS

- L153 - when presenting age, would suggest presenting whole numbers rather than decimals
- L153 – Need to write out “159” as it’s the start of a sentence
- L153 – suggest to not use “them” to describe study population, be specific about who you’re talking about even at the risk of sounding repetitive
- L154 – suggest changing “group” to “ages”
- L154 – need to write out “345” as it’s the start of a sentence
- L154 – suggest combining sentences about religion and ethnic groups
- L162 – again, suggest not to use “them”
- L162 – suggest including “n=255” in parentheses rather than part of main sentence structure
- L164 – suggest saying “174 women” or describing the percentage rather than simply “174” as it reads a bit dehumanizing
- L164 – again, suggest not using “them”
- L165 – need to write out “271”
- L165 – don’t use “them”
- L166 – need to write out “29”
- L166 – don’t use “them”
- L166 – did you query what kind of “problems” they faced or if they were specifically health related? This could be interpreted quite vaguely
- L167 – “289” what? Again, provide more clarity about *who* you’re speaking about
- L168 – “more than half” of whom?
- L168 – it’s not clear who “perceived threats” and “perceived severity” are the same thing, suggest providing more context here or earlier in the manuscript
- L168 – consider rephrase: “More than half (56.3%) of the study population noted that they perceived threats to their deliveries related to complications at the time of delivery.”
- L169 – consider rephrase: “the majority of pregnant women (89%) expressed that they perceived a benefit to giving birth at a health facility.”
- L170 – consider rephrase: “Approximately two thirds (n=278) of the women included in the study reported they felt they experienced barriers to giving birth at a health facility.”
- L171 – not overtly clear what is meant by “perceptions,” suggest adding clarity around what exactly is included in this
- L172 – important to interpret this data as well, what do the mean scores mean in context
- L180 – this is a run on sentence, consider rephrasing or listing these out, making sure to use congruent tenses for each element
- L189 – should be in past simple tense
- L192 – should be “were exposed”
- L195 – why is this being described as a “risk” when this is your desired outcome
- L197 – grammatically, women won’t “have” perceived threats”, they will “perceive threats” directly
- L199 – who are their counterparts? Be clear
- L200/201 – again, please review the grammar around “perceived benefits”
- L202 – suggested rephrase: “Accordingly, women who perceived benefits of giving birth at a health facility were 2.3 times more likely to also intend to deliver at a health facility.”
- L204 - who are their counterparts? Be clear
- L205 – consider rephrase: “Additionally, we found a negative association between perceived barriers of and intention of the pregnant women included in this study to give birth at a health facility.”
- L206/207 – strongly rephrasing sentence starting with “in view of that” to improve grammar and clarity
- Overall commentary: significant grammar adjustments, need for more specificity and clarity around presented results

DISCUSSION

- L216/217 – what are the risk assessment approaches? This hasn’t been introduced previously. Be sure to not present novel information in this section
- L217 – consider rephrase: “Births assisted by medical professionals in a healthcare setting are recognized as the best way to ensure safe and successful delivery outcomes for mother and baby.”
- L219 – do not use this section to present results. Instead interpret and compare to previous results, larger context
- L222 – this does not need to be a new paragraph. Work to combine information through the manuscript to reduce the number of new paragraphs
- L223/224/225 – I would suggest speaking to this information narratively rather than listing out percentages that aren’t particularly relevant to your argument
- L226 – seems to contract L221? Please clarify; again, suggested to not use percentages but instead explain narratively why this information is relevant
- L228/230 – suggest to combine these sentences
- L231 – suggest deleting “the study done…”
- L232 – suggest rephrase “unlike in previously published studies, our study was conducted in rural areas…”
- L233 – what do you mean by “far from evidence-based information due to numerous barriers”? unclear and vague
- L234 – delete “so”
- L236 – delete “those”
- L238 – rephrase “…more likely to intend to deliver…”
- L239/240 – combine last two sentences
- L244 – do not use this section to present results. Instead interpret and compare to previous results, larger context
- L246 – consider rephrase: “This result is in agreement with previous findings from Debremarkos…”
- L247/248 – suggest rephrasing to improve grammar and be more concise
- L249 – who is “they”? be clear about who you’re talking about
- L251 - do not use this section to present results. Instead interpret and compare to previous results, larger context
- L255 – consider rephrase “that means” to “Therefore, we can interpret that…”
- L257 - do not use this section to present results. Instead interpret and compare to previous results, larger context
- L261 – “would” doesn’t make sense here if you’re basing this statement off of findings
- L262 – “Furthermore, there are numerous socio-cultural and economic problems” this has not been discussed at all in this paper. Do not introduce new concepts without explanation. Consider deleting or adding more context throughout the manuscript
- L262/264 – accessibility/availability of healthcare facilities has also not been discussed previously in this manuscript
- L265 – need to further expand within limitations sections as this is not the only limitation
- Overall commentary: Need for significant grammar adjustments, do not present results in the discussion, need to use this section to tie results into context and larger body of existing work

CONCLUSION

- L268 – consider rephrasing conclusion section to improve grammar and flow. Additionally suggest using this section to call for future research or changes to practice
