# Supplementary material for: The intentions of pregnant women to give birth at a health facility and associated factors in the Aleta-Wondo rural District, Ethiopia: A community based cross-sectional study
Source: PLOS Glob Public Health. 2024 May 10;4(5):e0003215. doi: 10.1371/journal.pgph.0003215 (PMC11086849; doi:10.1371/journal.pgph.0003215)
Supplement: Attachment — Submitted filename: Response to Editor and Reviewers.docx [file pgph.0003215.s004.docx]

**Authors’ Point-by-point response to Editor’s and Reviewers’ comments**

We are very grateful to both the editor and reviewers for your constructive and valuable comments and suggestions. All the concerns raised so far will have a significant impact on improving the quality of our scholarly work. Appreciating your effort and valuable comments, we have provided possible reflections accordingly. Corrections with yellow color are for Reviewer#1 and green color is for Reviewer#2 in the revised manuscript with tack changes. Kindly find our reflections here. We hope you will consider the revised manuscript acceptable for publication in Plos Global Public Health Journal.

| **S.No** | | **Reviewer’s comments** | **Authors’ Response** |
| --- | --- | --- | --- |
| **Editor’s comments** | | | Dear editor, thank you for your feedback. We tried to address all the comments from the editor as well as Reviewers.  Concerns related with references and some overlapped information with previous work has been addressed. Kindly see our revised clean version of the manuscript. |
| **Reviewer#1** | | | |
| Overall comments to the author:   - Significant grammar adjustments necessary for publication, currently tone does match the caliber of journal selected - Flow seems very “piece-y” - important to provide additional clarity around methodology and why decisions were made - need for more specificity and clarity around presented results - do not present results in the discussion, need to use this section to tie results into context and larger body of existing work - Need to strengthen conclusion | | | Dear reviewer, thank you for your constructive comments and recommendation. We accepted your comments and revised all the bodies of the manuscript accordingly. Kindly see the clean version of the manuscript. |
|  | | ABSTRACT   - “out of the respondents…” – repeated 2x - “having information from health professionals…” – meaning not super clear overall, consider rephrase - “less than two-thirds of the respondents…” – repetitive, cut or rephrase | Thank you. We accepted your comments and revised the whole abstract to make it more precise and short information. Kindly see the clean version of the manuscript at **Page 2 Line 11-29.** |
| **INTRODUCTION** | | |  |
| 1. | | L32/33 – citation needed | Dear reviewer, thank you for your constructive comments.  We revised it accordingly. Kindly see at **page 3 line 31-33.** |
| 2. | | L34 – redundant | Dear reviewer, thank you for your constructive comments.  We revised it accordingly. |
| 3. | | L35 – “…can save nearly three-fourths this death.” Awkward phrasing, needs citation | Dear reviewer, thank you for your constructive comments.  We revised it accordingly. Kindly see at **page 3 line 33-34.** |
| 4. | | L37 – what are childbirth’s “courses”? awkward phrasing and unclear what is meant by this | Dear reviewer, thank you for your constructive comments.  We revised it accordingly. |
| 5. | | L40 – “are influencing” should be changed to “influence” to make the tenses match | Dear reviewer, thank again  We revised it accordingly. Kindly see at **page 3 line 41-44.** |
| 6. | | L42 – “despite agreement that…” this is arguably a political stance and is not in fact universal, consider speaking to the context of the study for a claim like this. You have yet to discuss the setting – this should really happen earlier on | Dear reviewer, thank you for your constructive comments.  Kindly see at **page 3 line 39-41.** |
| 7. | | L45/46 – citation needed | Dear reviewer, thank you for your constructive comments. We revised it accordingly. |
| 8. | | L46/47 – “In fact, need factors can be driven by pregnancy related factors such as perception” this does not make sense | Dear reviewer, thank you for your constructive comments. We revised it accordingly.  Kindly see the clean version of the manuscript. |
| 9. | | L47 – don’t use “in fact” twice in a row, rephrase | Dear reviewer, thank you for your constructive comments. We revised it accordingly. |
| 10. | | L47/48 – “…women’s intentions to involve in utilization of health facility…” awkward grammar | Dear reviewer, thank you for your constructive comments. We revised it accordingly. |
| 11. | | L51 – “in the world” consider rephrasing to say “worldwide” | Dear reviewer, thank you for your constructive comments. We revised it accordingly.  Kindly see the clean version of the manuscript. |
| 12. | | L51 – “highly varying…” consider rephrasing to say “highly variable” | Dear reviewer, thank you for your constructive comments. We revised it accordingly.  Kindly see the clean version of the manuscript. |
| 13. | | L52 – “poor” has not yet been defined, would suggest consistent use of WHO/World Bank terminology (e.g. low income, middle income, high income countries) | Dear reviewer, thank you for your constructive comments. We revised it accordingly.  Kindly see the clean version of the manuscript. |
| 14. | | L52/53 – suggest combining the first two sentences of this paragraph to be less repetitive and more concise | Dear reviewer, thank you for your constructive comments. We revised it accordingly.  Kindly see the clean version of the manuscript. |
| 15. | | L55 – a cause of maternal mortality being “high lifetime risk of maternal mortality” is not a particularly clear concept, consider rephrasing | Dear reviewer, thank you for your constructive comments. We revised it accordingly.  Kindly see the clean version of the manuscript. |
| 16. | | L56 – is this in Ethiopia specifically, worldwide or in SSA? Important to specify the context of the information you present as these will not be universal truths | Dear reviewer, thank you for your constructive comments. We revised it accordingly.  Kindly see the clean version of the manuscript, **page 3, line 48-51.** |
| 17. | | L56 – “poor health care seeking behavior/pregnant women’s ignorance of receiving skilled care” grammatically is clunky, also reads as quite judgmental and not helpful to the overall argument you’re trying to make, consider a rephrase | Dear reviewer, thank you for your constructive comments. We revised it accordingly.  Kindly see the clean version of the manuscript. |
| 18. | | L57 – potentially worth including information about what strategies have been utilized to date to strengthen your discussion | Dear reviewer, thank you for your constructive comments. We revised it accordingly.  Kindly see the clean version of the manuscript. |
| 19. | | L58 – “the practice of skilled birth attendance is still in alarming condition..” meaning unclear of this part of the sentence | Dear reviewer, thank you for your constructive comments. We revised it accordingly.  Kindly see the clean version of the manuscript. |
| 20. | | L60 – what model are you referencing? This is the first mention of a “model” in the paper, make sure you’re not skipping ahead or using different words to mean the same thing | Dear reviewer, thank you for your constructive comments. We accepted the comment and revised it. Kindly see the clean version of the manuscript. |
| 21. | | L61 – “some people...” too casual a tone for this sentence; overall meaning not clear, consider a rephrase | Dear reviewer, thank you for your constructive comments. We accepted the comment and revised it. Kindly see the clean version of the manuscript. |
| 22. | | L68 – what does this statement mean in context? Expected by whom? | Dear reviewer, thank you for your constructive comments. We accepted the comment and revised it. Kindly see the clean version of the manuscript. |
| 23. | | L71 – why must families consider that “every pregnancy” be at risk of the same complications when we know this isn’t the case? Suggest removing sentence as it does not support your argument | Dear reviewer, thank you for your constructive comments. We accepted the comment and revised it. Kindly see the clean version of the manuscript. |
| 24. | | L75 – delete “so” | Dear reviewer, thank you for your constructive comments. We accepted the comment and revised it. |
| 25. | | L76 – “yet, far it is not known…” already stated, consider rephrasing for grammar as well | Dear reviewer, thank you for your constructive comments. We accepted the comment and revised it. Kindly see the clean version of the manuscript. |
| 26. | | L78 – suggest providing more information about the exact context and why this was chosen, currently it seems like an afterthought | Dear reviewer, thank you for your constructive comments. We accepted the comment and revised it. Kindly see the clean version of the manuscript. |
| 27. | | Overall commentary: need to make significant grammar adjustments in this section, create more of a flow between ideas ensuring that point a leads to point b and so on, condense information to fit more of a paragraph format rather than one or two sentences at a time |  |
| **MATERIALS AND METHODS** | | |  |
| 28. | | L82 – delete “which is” | Dear reviewer, thank you for your constructive comments. We accepted the comment and revised it. |
| 29. | | L84 – it seems like you’re making a distinction between “female” and “women” which reads a bit odd, suggested rephrase: “…from which approximately 102,054 are female, with 44,039 of reproductive age.” | Dear reviewer, thank you for your constructive comments. We accepted the comment and revised it. Kindly see the clean version of the manuscript, page 5, line 67-72. |
| 30. | | L87 – why was it determined that this would be the methodology used? Provide justification in 1-2 sentences | Dear reviewer, thank you for your constructive comments. We accepted the comment and revised it. Kindly see the clean version of the manuscript. |
| 31. | | L87/88 – suggested rephrase “Pregnant women in the Aleta-Wondo rural district of Sidama Regional State were the population under study.” | Dear reviewer, thank you for your constructive comments. We accepted the comment and revised it. Kindly see the clean version of the manuscript, page 5, line 74-79. |
| 32. | | L88/89 – suggested rephrase: “Among this population pregnant women with…were included in this study analysis” | Dear reviewer, thank you for your constructive comments. We accepted the comment and revised it. Kindly see the clean version of the manuscript, page 5, line 74-79. |
| 33. | | L95 – why was this the assumed p value? I see that you’ve cited a source but not clear why it was chosen, be sure to justify | Dear reviewer, thank the comments. We accepted the comment and revised it. Kindly see the clean version of the manuscript, page 5, line 80-85. |
| 34. | | L96 – 421 what? You mention pregnant women and you mention kebeles, make sure you’re being very clear | Dear reviewer, thank you for your constructive comments. We accepted the comment and revised it. Kindly see the clean version of the manuscript, page 5, line page 5, line 80-89. |
| 35. | | L97 – important to have defined “kebeles” before this mention | Dear reviewer, thank you for your constructive comments. We accepted the comment and revised it. Kindly see the clean version of the manuscript. |
| 36. | | L97 – how was information about the kebeles collected? Public record, general knowledge, survey, etc – provide clarity | Dear reviewer, thank you for your constructive comments. We accepted the comment and revised it. Kindly see the clean version of the manuscript, page 5-6, line 83-87. |
| 37. | | L127 – suggested rephrase to improve tone “It was then translated back to English by translation specialists to verify the consistency of translation.”  Were these individuals part of the research team? Important to appropriately credit sources of support/information | Dear reviewer, thank you for your constructive comments. We accepted the comment and revised it. Kindly see the clean version of the manuscript. |
| 38. | | L129, L131 – not necessary to say “actual data collection,” you can directly say “data collection” | Dear reviewer, thank you for your constructive comments. We accepted the comment and revised it. Kindly see the clean version of the manuscript. |
| 39. | | L131 – did the pre-test verify the efficacy of the tool? Important to speak to the results of the pre-test if you’re going to mention it | Dear reviewer, thank you for your constructive comments. We accepted the comment and revised it. Kindly see the clean version of the manuscript. |
| 40. | | L132/133 – how were these checked? | Dear reviewer, thank you for your constructive comments. We accepted the comment and revised it. Kindly see the clean version of the manuscript. |
| 41. | | L137 – do not capitalize “Socio-demographic and Reproductive” | Dear reviewer, thank you for your constructive comments. We accepted the comment and revised it. Kindly see the clean version of the manuscript. |
| 42. | | Overall commentary: need to make significant grammar adjustments in this section, important to provide additional clarity around methodology and why decisions were made | Dear reviewer, thank you for your constructive comments. We accepted the comment and revised it. Kindly see the clean version of the manuscript. |
| 43. | | Do we have access to study materials/tools used? | Yes, on a reasonable condition. |
| **RESULTS** | | |  |
| 44. | | L153 - when presenting age, would suggest presenting whole numbers rather than decimals | Dear reviewer, thank you for your constructive comments. We accepted the comment and revised it. Kindly see the clean version of the manuscript, page 8, line 129-33. |
| 45. | | L153 – Need to write out “159” as it’s the start of a sentence | Dear reviewer, thank you for your constructive comments. We accepted the comment and revised it. Kindly see the clean version of the manuscript, page 8, line 129-33. |
| 46. | | L153 – suggest to not use “them” to describe study population, be specific about who you’re talking about even at the risk of sounding repetitive | Dear reviewer, thank you for your constructive comments. We accepted the comment and revised it. Kindly see the clean version of the manuscript, page 8, line 129-33. |
| 47. | | L154 – suggest changing “group” to “ages” | Dear reviewer, thank you for your constructive comments. We accepted the comment and revised it. Kindly see the clean version of the manuscript. |
| 48. | | L154 – need to write out “345” as it’s the start of a sentence | Dear reviewer, thank you for your constructive comments. We accepted the comment and revised it. Kindly see the clean version of the manuscript. |
| 49. | | L154 – suggest combining sentences about religion and ethnic groups | Dear reviewer, thank you for your constructive comments. We accepted the comment and revised it. Kindly see the clean version of the manuscript. |
| 50. | | L162 – again, suggest not to use “them” | Dear reviewer, thank you for your constructive comments. We accepted the comment and revised it. Kindly see the clean version of the manuscript. |
| 51. | | L164 – suggest saying “174 women” or describing the percentage rather than simply “174” as it reads a bit dehumanizing | Dear reviewer, thank you for your constructive comments. We accepted the comment and revised it. Kindly see the clean version of the manuscript. |
| 52. | | L164 – again, suggest not using “them” | Dear reviewer, thank you for your constructive comments. We accepted the comment and revised it. Kindly see the clean version of the manuscript. |
| 53. | | L165 – need to write out “271” |  |
| 54. | | L165 – don’t use “them” |  |
| 55. | | L166 – need to write out “29” |  |
| 56. | | L166 – don’t use “them” |  |
| 57. | | L166 – did you query what kind of “problems” they faced or if they were specifically health related? This could be interpreted quite vaguely |  |
| 58. | | L167 – “289” what? Again, provide more clarity about *who* you’re speaking about |  |
| 59. | | L168 – “more than half” of whom? | Dear reviewer, thank you for your constructive comments. We accepted the comment and revised it. Kindly see the clean version of the manuscript. |
| 60. | | L168 – it’s not clear who “perceived threats” and “perceived severity” are the same thing, suggest providing more context here or earlier in the manuscript |  |
| 61. | | L168 – consider rephrase: “More than half (56.3%) of the study population noted that they perceived threats to their deliveries related to complications at the time of delivery.” | Dear reviewer, thank you for your constructive comments. We accepted the comment and revised it. Kindly see the clean version of the manuscript, page 10, line 138-52. |
| 62. | | L169 – consider rephrase: “the majority of pregnant women (89%) expressed that they perceived a benefit to giving birth at a health facility.” |  |
| 63. | | L170 – consider rephrase: “Approximately two thirds (n=278) of the women included in the study reported they felt they experienced barriers to giving birth at a health facility.” |  |
| 64. | | L171 – not overtly clear what is meant by “perceptions,” suggest adding clarity around what exactly is included in this |  |
| 65. | | L172 – important to interpret this data as well, what do the mean scores mean in context | Dear reviewer, thank you for your constructive comments. We accepted the comment and revised it Kindly see the clean version of the manuscript, page 7, line 110-115. |
| 66. | | L180 – this is a run on sentence, consider rephrasing or listing these out, making sure to use congruent tenses for each element | Dear reviewer, thank you for your constructive comments. We accepted the comment and revised it Kindly see the clean version of the manuscript, page 12, line 159-80 |
| 67. | | L189 – should be in past simple tense |  |
| 68. | | L192 – should be “were exposed” |  |
| 69. | | L195 – why is this being described as a “risk” when this is your desired outcome |  |
| 70. | | L197 – grammatically, women won’t “have” perceived threats”, they will “perceive threats” directly |  |
| 71. | | L199 – who are their counterparts? Be clear |  |
| 72. | | L200/201 – again, please review the grammar around “perceived benefits” |  |
| 73. | | L202 – suggested rephrase: “Accordingly, women who perceived benefits of giving birth at a health facility were 2.3 times more likely to also intend to deliver at a health facility.” |  |
| 74. | | L204 - who are their counterparts? Be clear |  |
| 75. | | L205 – consider rephrase: “Additionally, we found a negative association between perceived barriers of and intention of the pregnant women included in this study to give birth at a health facility.” |  |
| 76. | | L206/207 – strongly rephrasing sentence starting with “in view of that” to improve grammar and clarity |  |
| 77. | | Overall commentary: significant grammar adjustments, need for more specificity and clarity around presented results | We accepted your comments and tried to revise the grammar adjustments significantly. Kindly see the clean version of the manuscript. |
| **DISCUSSION** | | |  |
| 78. | | L216/217 – what are the risk assessment approaches? This hasn’t been introduced previously. Be sure to not present novel information in this section | Dear reviewer, thank you for your constructive comments. We accepted the comment and revised it Kindly see the clean version of the manuscript, page 14, line 184-86 |
| 79. | | L217 – consider rephrase: “Births assisted by medical professionals in a healthcare setting are recognized as the best way to ensure safe and successful delivery outcomes for mother and baby.” |  |
| 80. | | L219 – do not use this section to present results. Instead interpret and compare to previous results, larger context |  |
| 81. | | L222 – this does not need to be a new paragraph. Work to combine information through the manuscript to reduce the number of new paragraphs | Dear reviewer, thank you for your constructive comments. We accepted the comment and revised it Kindly see the clean version of the manuscript. |
| 82. | | L223/224/225 – I would suggest speaking to this information narratively rather than listing out percentages that aren’t particularly relevant to your argument | Dear reviewer, thank you for your constructive comments. We accepted the comment and revised it Kindly see the clean version of the manuscript. |
| 83. | | L226 – seems to contract L221? Please clarify; again, suggested to not use percentages but instead explain narratively why this information is relevant | Dear reviewer, thank you for your constructive comments. We accepted the comment and revised it Kindly see the clean version of the manuscript. |
| 84. | | L228/230 – suggest to combine these sentences |  |
| 85. | | L231 – suggest deleting “the study done…” |  |
| 86. | | L232 – suggest rephrase “unlike in previously published studies, our study were conducted in rural areas…” | Dear reviewer, thank you for your constructive comments. We accepted the comment and revised it Kindly see the clean version of the manuscript. |
| 87. | | L233 – what do you mean by “far from evidence-based information due to numerous barriers”? unclear and vague | Dear reviewer, thank you for your constructive comments. We accepted the comment and revised it Kindly see the clean version of the manuscript. |
| 88. | | L234 – delete “so” | Dear reviewer, thank you for your constructive comments. We accepted the comment and revised it Kindly see the clean version of the manuscript. |
| 89. | | L236 – delete “those” |  |
| 90. | | L238 – rephrase “…more likely to intend to deliver…” |  |
| 91. | | L239/240 – combine last two sentences |  |
| 92. | | L244 – do not use this section to present results. Instead interpret and compare to previous results, larger context |  |
| 93. | | L246 – consider rephrase: “This result is in agreement with previous findings from Debremarkos…” |  |
| 94. | | L247/248 – suggest rephrasing to improve grammar and be more concise |  |
| 95. | | L249 – who is “they”? be clear about who you’re talking about |  |
| 96. | | L251 - do not use this section to present results. Instead interpret and compare to previous results, larger context | Dear reviewer, thank you for your constructive comments. We accepted the comment and revised it Kindly see the clean version of the manuscript. |
| 97. | | L255 – consider rephrase “that means” to “Therefore, we can interpret that…” |  |
| 98. | | L257 - do not use this section to present results. Instead interpret and compare to previous results, larger context |  |
| 99. | | L261 – “would” doesn’t make sense here if you’re basing this statement off of findings |  |
| 100. | | L262 – “Furthermore, there are numerous socio-cultural and economic problems” this has not been discussed at all in this paper. Do not introduce new concepts without explanation. Consider deleting or adding more context throughout the manuscript |  |
| 101. | | L262/264 – accessibility/availability of healthcare facilities has also not been discussed previously in this manuscript |  |
| 102. | | L265 – need to further expand within limitations sections as this is not the only limitation |  |
| 103. | | Overall commentary: Need for significant grammar adjustments, do not present results in the discussion, need to use this section to tie results into context and larger body of existing work | We accepted your comments and tried to revise the grammar adjustments significantly. Kindly see the clean version of the manuscript. |
| **CONCLUSION** | | |  |
| 104. | | L268 – consider rephrasing conclusion section to improve grammar and flow. Additionally suggest using this section to call for future research or changes to practice | We accepted your comments and tried to revise the grammar adjustments significantly. Kindly see the clean version of the manuscript, page 16, line 226-33. |
| **Reviewer#2** | | | |
| 1 | Unnecessary repetition of sentence regarding intention to give birth at health facility in abstract part line 22-23…..why? | | Thank you. We accepted your comments and revised the whole abstract to make it more precise and short information. Kindly see the clean version of the manuscript. |
| 2 | Threat perception is emphasized in this model as a key step in distinguishing the importance of taking a recommended action. See line 61-62 page 4. Which model? | | Dear reviewer, thank you for your constructive comments. We accepted the comment and revised it. Kindly see the clean version of the manuscript. |
| 3 | What was the research gap regarding intention of women to give birth at health facility? If any put your strong justification in final paragraph of the background. | | We accepted your comments and tried to revise the manuscript. Kindly see the clean version of the manuscript, page 4, line 58-64. |
| 4 | Describe the characteristics of study population regarding institutional delivery and health facility availability/accessibility and other important information in your study setting. See page 5 line 82-85. | | We accepted your comments and tried to revise manuscript accordingly. Kindly see the clean version of the manuscript, page 5, line 67-72.. |
| 5 | Specify study period (starting and end date). You said “This study was conducted from January to February 2022”. See page 5 line 85. | | We accepted your comments and tried to revise manuscript accordingly. Kindly see the clean version of the manuscript, page 5, line 71-72. |
| 6 | Revise this sentence: Whereas, the study population was pregnant women who were living in the randomly selected kebeles, and fulfilled  the eligibility criteria were. See Line 89-90 | | We accepted your comments and tried to revise manuscript accordingly. Kindly see the clean version of the manuscript. |
| 7 | You said “An interviewer-administered, structured, and pretested questionnaire was developed by reviewing related literature to collect data”. What are those related literature? See line 107. | | Dear Reviewer, We accepted your comments and tried to revise manuscript accordingly. Kindly see the clean version of the manuscript. |
| 8 | The dependent variables were Socio-demographic and Reproductive and health service related characteristics of the study participants. Revise it. See line 137-138 | | We accepted your comments and tried to revise manuscript accordingly. Kindly see the clean version of the manuscript. |
| 9 | There was a problem of organizing your manuscript with appropriate sequence. For example you organized study variables and data quality control after data analysis. Revise it. | | Dear Reviewer, We accepted your comments and tried to revise manuscript accordingly. Kindly see the clean version of the manuscript. |
| 10 | Have you checked multi-collinearity of independent variables? | | Yes, Kindly see the clean version of the manuscript. |
| 11 | Approval number at which responsible individual in college provided? | | Yes, Kindly see the clean version of the manuscript. |
| 12 | Page 9... mention others for religion and ethnicity | | We accepted your comments and tried to revise manuscript accordingly. Kindly see the clean version of the manuscript. |
| 13 | If you were intended to use health belief model why you missed other constructs of this model? | |  |
| 14 | You said “Facing different challenges would affect pregnant woman’s decision-making ability to use the available services. Furthermore, there are numerous socio-cultural and economic problems”. See discussion part line 261. But you haven’t addressed economic related factors (income/wealth index of household) and cultural factors (qualitative study). .. | |  |
|  | State your strength of study if any | | We accepted your comments and tried to revise manuscript accordingly. Kindly see the clean version of the manuscript. |
|  | Some references are too old (see reference number 2,3 10,11,19) | | We accepted your comments and tried to revise it accordingly. Kindly see the clean version of the manuscript. |
|  | **Overall comment**  Manuscript was well written and the problem under the study was interesting public health issue. My concern was justification for this study because there were studies done regarding this issue. Some editorial, topographic and description problem should be corrected. | | Dear reviewer we would like to say thank you for your scientific comments and suggestions. We accepted all your comments and tried to address them. |
