# Supplementary material for: The intentions of pregnant women to give birth at a health facility and associated factors in the Aleta-Wondo rural District, Ethiopia: A community based cross-sectional study
Source: PLOS Glob Public Health. 2024 May 10;4(5):e0003215. doi: 10.1371/journal.pgph.0003215 (PMC11086849; doi:10.1371/journal.pgph.0003215)
Supplement: Attachment — Submitted filename: Reviewer comment to author JPGPH.docx [file pgph.0003215.s003.docx]

**Reviewer comment to author**

1. [The study presents the results of primary scientific research](https://journals.plos.org/globalpublichealth/s/criteria-for-publication#loc-1). -**yes**
2. [Results reported have not been published elsewhere](https://journals.plos.org/globalpublichealth/s/criteria-for-publication#loc-2).**-yes**
3. [Experiments, statistics, and other analyses are performed to a high technical standard and are described in sufficient detail.](https://journals.plos.org/globalpublichealth/s/criteria-for-publication#loc-3)-**yes**
4. [Conclusions are presented in an appropriate fashion and are supported by the data](https://journals.plos.org/globalpublichealth/s/criteria-for-publication#loc-4)-**yes**.
5. [The article is presented in an intelligible fashion and is written in standard English](https://journals.plos.org/globalpublichealth/s/criteria-for-publication#loc-5)-**moderate/requires further modification.**
6. [The research meets all applicable standards for the ethics of experimentation and research integrity](https://journals.plos.org/globalpublichealth/s/criteria-for-publication#loc-6)-**yes/requires some details in ethical clearance regarding approval letter and types of the consent obtained.**
7. [The article adheres to appropriate reporting guidelines and community standards for data availability](https://journals.plos.org/globalpublichealth/s/criteria-for-publication#loc-7)-**yes/but authors must submit STROBE checklist for cross sectional study.**

General comments

- Unnecessary repetition of sentence regarding intention to give birth at health facility in abstract part line 22-23…..why?
- Threat perception is emphasized in this model as a key step in distinguishing the importance of taking a recommended action. See line 61-62 page 4. Which model?
- What was the research gap regarding intention of women to give birth at health facility? If any put your strong justification in final paragraph of the background.
- Describe the characteristics of study population regarding institutional delivery and health facility availability/accessibility and other important information in your study setting. See page 5 line 82-85.
- Specify study period (starting and end date). You said “This study was conducted from January to February 2022”. See page 5 line 85.
- Revise this sentence: Whereas, the study population was pregnant women who were living in the randomly selected kebeles, and fulfilled
   the eligibility criteria were. See Line 89-90
- You said “An interviewer-administered, structured, and pretested questionnaire was developed by reviewing related literature to collect data”. What are those related literature? See line 107.
- The dependent variables were Socio-demographic and Reproductive and health service related characteristics of the study participants. Revise it. See line 137-138
- There was a problem of organizing your manuscript with appropriate sequence. For example you organized study variables and data quality control after data analysis. Revise it.
- Have you checked multi-collinearity of independent variables?
- Approval number at which responsible individual in college provided?
- Page 9... mention others for religion and ethnicity
- If you were intended to use health belief model why you missed other constructs of this model?
- You said “Facing different challenges would affect pregnant woman’s decision-making ability to use the available services. Furthermore, there are numerous socio-cultural and economic problems”. See discussion part line 261. But you haven’t addressed economic related factors (income/wealth index of household) and cultural factors (qualitative study). ..
- State your strength of study if any
- Some references are too old (see reference number 2,3 10,11,19)

**Overall comment**

Manuscript was well written and the problem under the study was interesting public health issue. My concern was justification for this study because there were studies done regarding this issue. Some editorial, topographic and description problem should be corrected.

**Final decision-** Accept with minor modification.
